# Virulence and antifungal susceptibility of microsatellite genotypes of Candida albicans from superficial and deep locations

**DOI:** 10.1002/yea.3397

**Published:** 2019-06-13

**Authors:** Meizhu Wang, Yu Cao, Maoning Xia, Abdullah M.S. Al‐Hatmi, Weizheng Ou, Yanyan Wang, Andriy A. Sibirny, Liang Zhao, Chenggang Zou, Wanqing Liao, Fengyan Bai, Xie Zhi, Sybren de Hoog, Yingqian Kang

**Affiliations:** ^1^ Key Laboratory of Environmental Pollution Monitoring and Disease Control, Ministry of Education of Guizhou & Guizhou Talent Base for Microbiology and Human Health, Key Laboratory of Medical Microbiology and Parasitology of Education Department of Guizhou, School of Basic Medical Sciences Guizhou Medical University Guiyang China; ^2^ School of Big Health Guizhou Medical University Guiyang China; ^3^ Westerdijk Fungal Biodiversity Institute Utrecht The Netherlands; ^4^ Department of Clinical Laboratory People's Hospital of Dazu District Chongqing China; ^5^ Ministry of Health Directorate General of Health Services Ibri Oman; ^6^ Department of Clinical Lab Guiyang Public Health Treatment Center Guiyang China; ^7^ Infection Control Section The Affiliated Hospital of Guizhou Medical University Guiyang China; ^8^ Department of Biotechnology and Microbiology University of Rzeszow Rzeszow Poland; ^9^ Institute of Cell Biology NAS of Ukraine Lviv Ukraine; ^10^ State Key Laboratory for Conservation and Utilization of Bioresources in Yunnan Yunnan University Kunming China; ^11^ Shanghai Key Laboratory of Molecular Medical Mycology, Department of Dermatology, Shanghai Institute of Medical Mycology, Changzheng Hospital Second Military Medical University Shanghai China; ^12^ Systematic Mycology and Lichenology Laboratory, Institute of Microbiology Chinese Academy of Sciences Beijing China; ^13^ Department of Dermatology People's Hospital of Guangxi Zhuang Autonomous Region Nanning China; ^14^ Centre of Expertise in Mycology of Radboudumc/Canisius Wilhelmina Hospital Nijmegen The Netherlands

**Keywords:** 25S rDNA genotype, antifungal susceptibility, CAI microsatellite genotype, Candida albicans, urothelial carcinoma

## Abstract

A set of 185 strains of Candida albicans from patients with vulvovaginal candidiasis (VVC) and from non‐VVC clinical sources in southwest China was analysed. Strains were subjected to genotyping using CAI microsatellite typing and amplification of an intron‐containing region of the 25S rRNA gene. Microsatellite genotypes of strains from non‐VVC sources showed high polymorphism, whereas those of VVC were dominated by few, closely similar genotypes. However, among non‐VVC strains, two genotypes were particularly prevalent in patients with lung cancer. 25S rDNA genotype A was dominant in VVC sources (86.7%), whereas genotypes A, B, and C were rather evenly distributed among non‐VVC sources; known genotypes D and E were not found. In an experimental mouse model, isolates from lung cancer and AIDS patients proved to have higher virulence than VVC strains. Among 156 mice infected with C. albicans, 19 developed non‐invasive urothelial carcinoma. No correlation could be established between parameters of virulence, source of infection, and incidence of carcinoma. C. albicans strains from VVC were less susceptible to itraconazole than the strains from non‐VVC sources, whereas there was small difference in antifungal susceptibility between different 25S rDNA genotypes of C. albicans tested against amphotericin B, itraconazole, fluconazole, and flucytosine.

## 1 INTRODUCTION

Candida albicans is involved in a wide variety of infections, ranging from mucosal or vaginal disorders in generally healthy persons to life‐threatening, systemic infections in individuals with severely impaired immunity (Achkar & Fries, 2010). Mucocutaneous candidiasis can be divided into genitourinary and non‐genital disease. Vulvovaginal candidiasis (VVC) is one of the most frequent manifestations (de Repentigny, Lewandowski, & Jolicoeur, 2004). C. albicans expresses successful adaptation to the human body in response to various stimuli, being able to sustain as a commensal colonizer and as an opportunistic pathogen (Egbe, Paget, Wang, & Ashe, 2015). Not only the host's immune system is a major factor balancing the transition from commensalism to pathogenicity, but also fungal features as adhesion, hyphal formation, phenotypic switching, extracellular enzyme production, and biofilm formation contribute to C. albicans virulence. Selective proliferation of some genes of commensal strains may precede the transition of C. albicans to infection (Abdulrahim, McManus, Flint, & Coleman, 2013; da Silva‐Rocha, Lemos, Svidizisnki, Milan, & Chaves, 2014). The possibility that chronic inflammation, in which C. albicans infection can play a role, may enhance the development of cancer has been brought forward by several authors (e.g., Norgaard, Thomsen, Farkas, Mogensen, & Sorensen, 2013; Ramirez‐Garcia et al., 2016).

Epidemiological genotyping is relevant in the context of species prevalence, for elucidation of virulence factors and mechanisms of drug resistance, and is of practical importance to support treatment options in candidiasis. Molecular studies have shown that C. albicans isolates exhibit a high level of genetic diversity. Therefore, reproducible and precise genotyping methods are required, illustrating possible relationships between strain genotypes, phenotypic properties, and host interactions. Strains may differ considerably in virulence, antifungal susceptibility, and other clinically relevant parameters, and appropriate genotyping methods may reveal microevolution by detecting small genetic variations indicating adaptation to long‐term host response. In recent years, short tandem repeats or microsatellites have been used as molecular markers for population genetics and genotyping. Microsatellite loci (e.g., CAI, CAIII, CAV, CAVI, and CAVII) located in coding or non‐coding regions have been employed for strain typing of C. albicans (Sampaio et al., 2003, 2005). GeneScan analyses of the CAI microsatellites proved to be particularly powerful (Bai, 2014; Carvalho‐Pereira, Vaz, Carneiro, Pais, & Sampaio, 2015). Previous studies showed promising results when using this method to determine genetic diversity in C. albicans with VVC and non‐VVC clinical features (Bai, 2014; Fan et al., 2008; Ge et al., 2010). The method proved to be sufficiently sensitive to reveal consistent geographical structuring within the species.

The main aim of our study is to apply genotyping using GeneScan analysis of CAI loci aiming to compare genetic diversities of C. albicans isolates from vaginal candidiasis with those from other clinical sources in patients from the same region. Experimental mouse infection is used to establish comparative virulence of strains from particular patient groups, and in vitro susceptibility is tested to reveal eventual correlations between virulence, source of infection, drug resistance, and genotype.

## 2 MATERIALS AND METHODS

### 2.1 Collection and identification of test isolates

A total of 185 independent C. albicans strains were isolated from patients treated in the Affiliated Hospital of Guizhou Medical University (Table S1), the Guiyang Women and Children's Healthcare Hospital, the No. 2 Affiliated Hospital of Guiyang College of Traditional Chinese Medicine, the Baiyun No. 1 People's Hospital, and the Guiyang No. 5 People's Hospital. The set included 90 strains from high vaginal swabs of female patients with vaginal candidiasis and 95 strains from bronchoalveolar lavage fluid, urine, and blood from cases of systemic candidiasis of adult male and female patients with various types of candidiasis. C. albicans ATCC 90028 (=CBS 8837) was used as reference. For animal experiments, nine clinical strains from symptomatic patients and three from asymptomatic hosts (Table 1) and reference strain ATCC 10231 (=CBS 6431) were used. All isolates were maintained in cryopreservation in 20% glycerol (wt/wt) at −AT°C.

**Table 1:** Occurrence of carcinoma in mice infected with Candida albicans

Group	No.	CAI genotype	Source	30–60 days	61–90 days	91–120 days	121–168 days	Number of carcinomas	Percentage incidence
1	GYF006	30–45	VVC	L(1)				1	8.3
2	GYZ249	21–21	Lung cancer	L(1)			L(1)/H(2)	4	33.3
3	ATCC10231	Untested	Bronchopneumonia			H(1)		1	8.3
4	GYWu27	21–21	HIV/AIDS					0	0.0
5	GYWu45	21–21	HIV/AIDS	L(1)			H(1)	2	16.7
6	GYF76	32–46	VVC				H(1)	1	8.3
7	GYWu20	21–33	HIV/AIDS		H(1)			1	8.3
8	GYF091	30–46	VVC	L(1)/H(1)				2	16.7
9	GYZ210	21–33	Lung cancer			H(1)	H(1)	2	16.7
10	GYZ072	21–33	Lung cancer		H(1)			1	8.3
11	BK60	Untested	Healthy host				H(1)	1	8.3
12	S165	Untested	Healthy host			H(1)	H(1)	2	16.7
13	1107	Untested	Healthy host				H(1)	1	8.3
14	Saline	‐	‐					0	0.0

*Note*. H, non‐invasive urothelial carcinoma, high grade; L, non‐invasive urothelial carcinoma, low grade.

Abbreviation: VVC, vulvovaginal candidiasis.

### 2.2 DNA extraction and polymerase chain reaction

Nuclear DNA was extracted according to methods described by Kaiser, Michaelis, Mitchell, and Laboratory (1994). The rDNA ITS region was amplified using primers ITS1 and ITS4 (Kageyama et al., 2004). Obtained sequence data were compared with those published in GenBank using BLAST. The CAI microsatellite locus was amplified by with primers 5′‐ATG CCA TTG AGT GGA ATT GG‐3′ (fw) and 5′‐AGT GGC TTG TGT TGG GTT TT‐3′ (rv) according to Sampaio et al. (2005). For GeneScan analysis, the forward primer was 5′ fluorescently labelled with 6‐carboxyfluorescein. Polymerase chain reaction (PCR) amplification was performed in a thermocycler (iCycler, Bio‐Rad, Hercules, CA, USA) with initial denaturation at 95°C for 5 min, 33 cycles of denaturation at 94°C for 30 s, annealing at 60°C for 30 s, and extension at 72°C for 1 min and a final extension step of 7 min at 72°C. Fragment sizes of amplified DNA were determined automatically by GeneScan software V3.7. CAI genotypes were designated by the number of trinucleotide repeat units in both alleles (because of the diploid nature of C. albicans) of the microsatellite locus, as described by Sampaio et al. (2005). Primer pairs spanning the transposable intron in the 25S rDNA as follows: CA‐INT‐L (5′‐ATA AGG GAA GTC GGC AAA ATA GAT CCG TAA‐3′) and CA‐INT‐R (5′‐CCT TGG CTG TGG TTT CGC TAG ATA GTA GAT‐3′). The PCR conditions were denaturation for 3 min at 94°C prior to 30 cycles of 94°C for 1 min, 65°C for 1 min, and 72°C for 2.5 min and a final extension at 72°C for 10 min.

### 2.3 Antifungal susceptibility

In vitro susceptibilities of *Candida* strains obtained from blood samples were tested against four antifungal agents: fluconazole (0.12–64 μg/ml), itraconazole (0.015–16 μg/ml), amphotericin B (0.03–16 μg/ml), and flucytosine (0.12–64 μg/ml). MICs were determined by broth microdilution with a commercial frozen plate for antifungal susceptibility testing (Eiken Chemical Co., Shanghai, China) that complied with Clinical Laboratory and Standards Institute (CLSI) guideline M27‐A3, following the manufacturer's instructions. MIC values were interpreted according to the criteria in CLSI guideline M27‐S4 and the epidemiological cut‐off value (Pfaller & Diekema, 2012). C. albicans IFM 40213 (=ATCC 90028) and Candida parapsilosis ATCC 22019 were used as quality control isolates. We evaluated whether the MIC/MEC values correlated with different genotypes using the Mann–Whitney–Wilcoxon test for skewed distribution.

### 2.4 Mouse model of disseminated candidiasis

Twelve isolates were used in an experimental mouse model of hematogenously disseminated candidiasis as described previously by Sampaio et al. (2010). Nine of these originated from symptomatic and three from healthy individuals; ATCC 10231 = CBS 6431 was applied as reference. The set included genotypes 21–33 and 21–21, which were primarily derived from patients with lung cancer or AIDS, whereas 30–45, 32–46, and 30–46 were the dominant genotypes of VVC. According to the principle of randomization, control and double‐blind strains and saline were divided into 14 groups (12 mice for each group; Table 1). Strains analysed (Table S1) originated from unrelated patients in different hospitals. The study was conducted under the guidelines and approval of the Research Ethics Committee of Guizhou Medical University. Briefly, 156 female KM mice (18‐ to 22‐g body weight; 6 weeks, Guizhou Medical University) were injected with 5 × 10^5^ cells of the corresponding strain and via the lateral tail vein (Amorim‐Vaz, Delarze, Ischer, Sanglard, & Coste, 2015; Taylor et al., 2000), and 12 mice in the control group were injected with the same volume of saline. All inoculums were confirmed by quantitative culture. For preparation of inoculums, thawed cells from the original stock were grown on PDA medium at 37°C. In each experiment, all isolates were tested simultaneously, and inocula were confirmed by colony‐forming unit counting of the suspensions used to infect the mice. Animal welfare was assessed twice daily during 5 months. The mice were monitored at least two times daily, and dead mice were dissected immediately. Brain, liver, spleen, and kidneys from each mouse were harvested and weighed. After 5 months of infection, all mice were sacrificed for anatomical examination.

### 2.5 Histopathology

Organs excised from all infected mice were fixed in 10% phosphate‐buffered formalin, embedded in paraffin, sectioned, and stained with haematoxylin–eosin (H&E) according to Kumar, Saraswat, Tati, and Edgerton (2015). On the basis of 1,000 neoplastic nuclei, Ki‐67 labelling index was calculated in each slide as the percentage of immunopositive nuclei (Šteňo et al., 2014).

### 2.6 Statistics

Survival of mice was compared with log‐rank test and Gehan–Breslow–Wilcoxon test, using GraphPad Prism 7 for Windows. *P* values ≤.05 were considered to be significant. 25S rDNA group I intron genotypes for C. albicans from different sources and carcinoma of mice in each group were compared using chi square (Li, 2010). PowerMarker V3.25 software was used for calculation of polymorphism information content and Nei's genetic distance of CAI gene. Construction of a distance tree was done using clustering with the unweighted pair group method with arithmetic means. We generated odds ratio (OR) for associations between VVC and VVC‐prevalent CAI genotypes using generalized estimating unconditional logistic regression.

## 3 RESULTS

### 3.1 Genotyping

A total of 185 independent isolates of *Candida* were identified down to species level using the rDNA ITS spacer region; all isolates were confirmed to be C. albicans. The strains were analysed with 25S rDNA amplification, of which 122 were classified as genotype A (65.9%), 35 were classified as genotype B (18.9%), and 28 were classified as genotype C (15.1%). Regarding the 90 strains isolated from VVC, genotype A was preponderant with 78 isolates (86.7%); seven isolates were genotype B (7.8%), and five isolates were genotype C (5.6%). For the strains isolated from non‐VVC, there were 44 isolates with genotype A (46.3%), 28 isolates with genotype B (29.5%), and 23 isolates with genotype C (24.2%). Data were inspected with *χ* ^2^ (*p* < .05) in the Statistical Package for the Social Sciences V10.0 software. Results showed significantly different proportions in 25S rDNA group I intron between the strains from VVC and non‐VVC (0.000).

Ninety strains isolated from female patients with vaginal candidiasis (VVC) and 95 from other clinical sources were genotyped for the CAI region. In the 95 non‐VVC C. albicans strains, PCR products revealed 24 separate alleles consisting of fragments with different lengths, varying between 189 bp (11 repeats) and 291 bp (45 repeats), and 40 distinct genotypes were recognized. In the 90 VVC strains, we identified a total of 22 separate alleles and 27 distinct genotypes, and the length of CAI fragments varied between 189 bp (11 repeats) and 297 bp (47 repeats). Seven genotypes are reported here for the first time.

**Figure 1:**
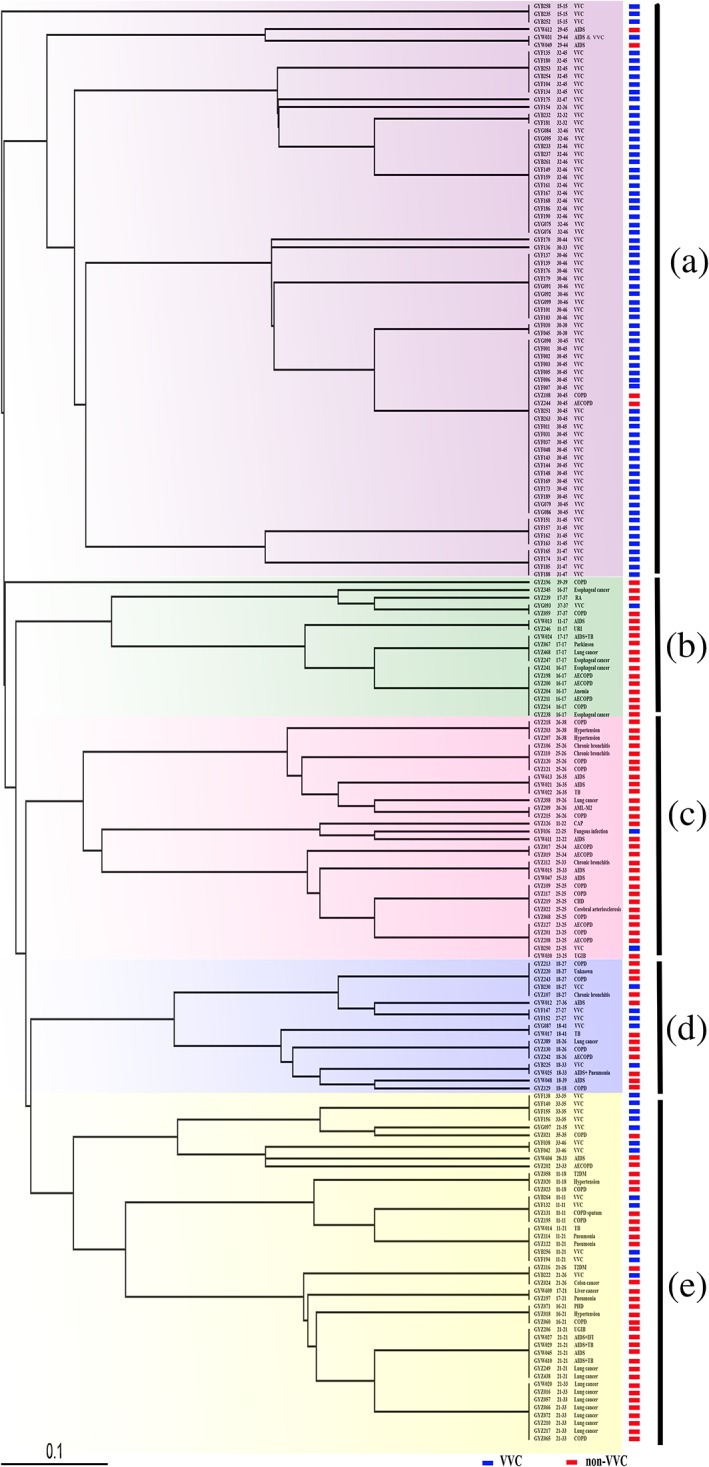
Construction of an unweighted pair group method with arithmetic means distance tree of CAI gene with 185 Candida albicans strains isolated from vulvovaginal candidiasis (VVC) and non‐VVC. The blue rectangle‐marked strains are isolated from VVC; and red rectangle marked strains are isolated from non‐VVC. COPD: chronic obstructive pulmonary disease; AECOPD: acute exacerbation of chronic obstructive pulmonary disease; AIDS: acquired immunodeficiency syndrome; RA: rheumatoid arthritis; URI: upper respiratory tract infection; TB: tuberculosis; AML‐M2: acute myeloblastic leukaemia with differentiation; CAP: community‐acquired pneumonia; CHD: congenital heart defect; UGIB: upper gastrointestinal bleeding; T2DM: diabetes mellitus type 2 [Colour figure can be viewed at wileyonlinelibrary.com]

The 185 clinical isolates of C. albicans were divided into five clusters by CAI microsatellite polymorphism analysis (Figure 1). Most of the genotypes of the VVC strains belong to cluster A (70/90, 77.8%). Prevalent genotypes among VVC strains in cluster A are 30–45 (23 strains, 31.9%), followed by 32–46 (14 strains, 19.4%) and 30–46 (nine strains, 12.5%). These three common genotypes and another seven similar genotypes account for 63 strains (70.0%) and show a wide distribution. Unconditional logistic regression analysis showed that the risk OR of prevalent CAI genotypes with VVC was 4.3. However, CAI genotypes of non‐VVC strains are mainly found widely distributed in clusters B, C, D, and E. Among non‐VVC strains, 10 stains, seven of which belonged to the closely similar genotypes 21–33 and 21–21, were associated with lung cancer.

### 3.2 Antifungal susceptibility

Table 2 summarizes the in vitro susceptibilities of 90 VVC and 59 non‐VVC isolates of C. albicans to amphotericin B, itraconazole, fluconazole, and flucytosine. The data are presented as MICs, MIC ranges, median MIC, MIC_50_, and MIC_90_. The analysis using CLSI methodology demonstrates that amphotericin B spanned a narrow range of 0.125–1 with GM MIC of 0.70 μg/ml for VVC and a MIC range of 0.25–1 with GM MIC 0.71 μg/ml for non‐VVC. The MIC_50_ and MIC_90_ for amphotericin B were 1 μg/ml for both VVC and non‐VVC. The GM MICs for 5‐flucytosine for VVC and non‐VVC, respectively, were 1.77 and 0.18 μg/ml, the MIC_50_ for flucytosine was 2 and 0.125 μg/ml, and the MIC_90_ for flucytosine was 4 and 0.25 μg/ml. For fluconazole, the GM MIC, MIC range, MIC_50_, and MIC_90_ were 1.71, 0.06–64, 2, and 8 μg/ml for VVC and 0.91, 0.125 to >64, 0.5, and 4 μg/ml for non‐VVC. For itraconazole, the GM MIC, MIC range, MIC_50_, and MIC_90_ were 0.39, 0.03–8, 0.5, and 2 for VVC and 0.22, 0.015 to >8, 0.125, and 8 μg/ml for non‐VVC. Overall, all of the strains were susceptible to amphotericin B. There was no significant difference of VVC and non‐VVC in susceptibility to 5‐flucytosine (.985) and fluconazole (.272). However, C. albicans from VVC strains were less susceptible to itraconazole than the strains from non‐VVC sources (0.000).

**Table 2:** Results of four antifungal drugs against Candida albicans from VVC and non‐VVC

Antifungal	MIC (μg/ml)	VVC	Non‐VVC
Amphotericin B	MIC range	0.125–1	0.25–1
	GM MIC	0.70	0.71
	MIC_50_	1	1
	MIC_90_	1	1
5‐Fluorocytosine	MIC range	0.25–64	<0.125 to >64
	GM MIC	1.77	0.18
	MIC_50_	2	0.125
	MIC_90_	4	0.25
Fluconazole	MIC range	0.06–64	0.125 to >64
	GM MIC	1.71	0.91
	MIC_50_	2	0.5
	MIC_90_	8	4
Itraconazole	MIC range	0.03–8	<0.015 to >8
	GM MIC	0.39	0.22
	MIC_50_	0.5	0.125
	MIC_90_	2	8

Abbreviation: VVC, vulvovaginal candidiasis.

Table 3 shows the analysis of the MIC ranges, median MIC, MIC_50_, and MIC_90_ of C. albicans different 25S rDNA genotypes (A, B, and C). All strains were susceptible to amphotericin B; there was no significant difference in susceptibility to remaining drugs tested (.315, .391, and .409, respectively). Genotypes were differentially susceptible to flucytosine and fluconazole. The C. albicans 25S rDNA genotypes of group A were considerably less susceptible to fluconazole, flucytosine, and itraconazole than genotypes B and C. MIC_50_ and MIC_90_ were 2 and 4 μg/ml for flucytosine and 2 and 8 μg/ml for fluconazole, respectively.

**Table 3:** Result of antifungal drugs sensitivity test of Candida albicans different 25S rDNA genotypes

Antifungal agents	MIC	Genotype A	Genotype B	Genotype C
Amphotericin B	MIC range (ug/ml)	0.125–1	0.25–1	0.125–1
	GM MIC (ug/ml)	0.70	0.76	0.70
	MIC_50_	1	1	1
	MIC_90_	1	1	1
5‐Fluorocytosine	MIC range (ug/ml)	<0.125 to >64	<0.125–1	<0.125–0.5
	GM MIC (ug/ml)	1.22	0.17	0.17
	MIC_50_	2	0.125	0.125
	MIC_90_	4	0.5	0.5
Fluconazole	MIC range (ug/ml)	0.06 to >64	0.125 to >64	0.25 to >64
	GM MIC (ug/ml)	1.25	1.06	2.00
	MIC_50_	1	0.5	2
	MIC_90_	8	16	64
Itraconazole	MIC range (ug/ml)	<0.015 to >8	0.03 to >8	0.03 to >8
	GM MIC (ug/ml)	0.30	0.21	0.50
	MIC_50_	0.25	0.125	0.25
	MIC_90_	2	8	8

### 3.3 Murine model

**Figure 2:**
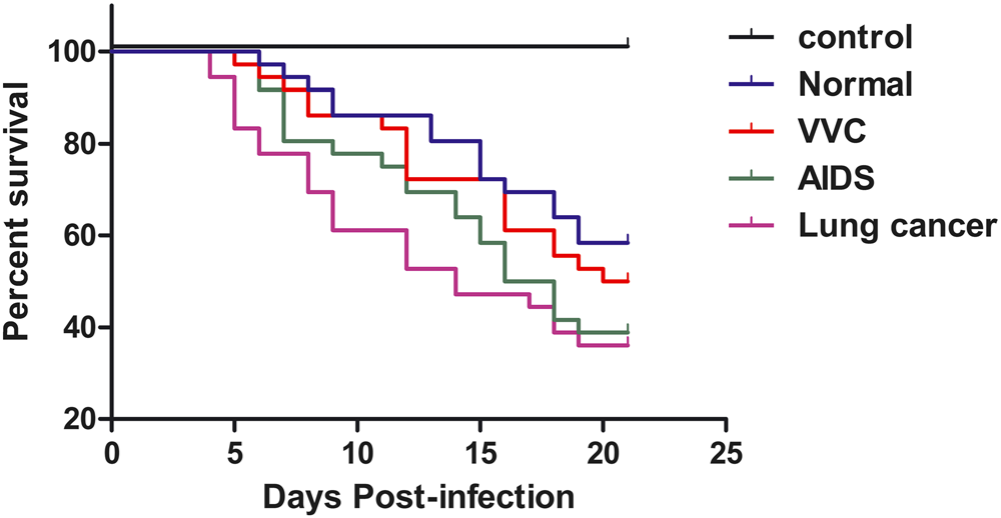
Survival curves of KM mice following infection with Candida albicans strains. Mice were inoculated i.v. with 5 × 10^5^ cells of strains from lung cancer, HIV/AIDS, VVC, and normal (asymptomatic) individuals; conditions of mice were assessed daily for 21 days [Colour figure can be viewed at wileyonlinelibrary.com]

**Figure 3:**
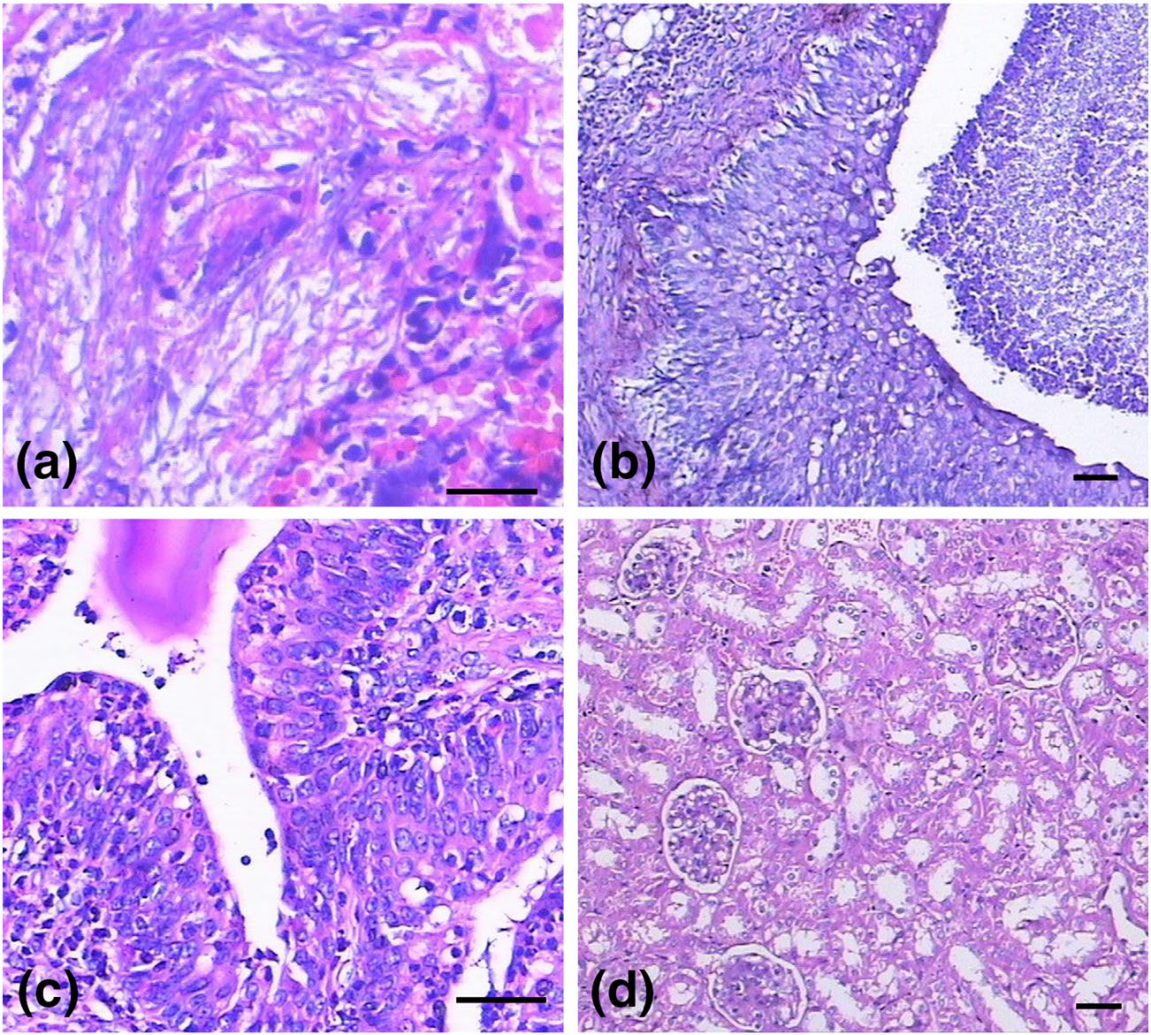
Pathological changes in the kidney of the mice infected with Candida albicans (HE staining, A × 400, B × 200, C × 400, D × 200). (a) Kidney section of mouse (died on Day 48) inoculated by GYW045 of genotype 21–21; (b) kidney section of mouse (died on Day 67) inoculated by GYZ210 of genotype 21–33; (c) kidney sections of mouse (euthanized on Day 168) inoculated by GYZ249 of genotype 21–21; (d) kidney section of normal mouse (scale bar = 50 μm) [Colour figure can be viewed at wileyonlinelibrary.com]

**Figure 4:**
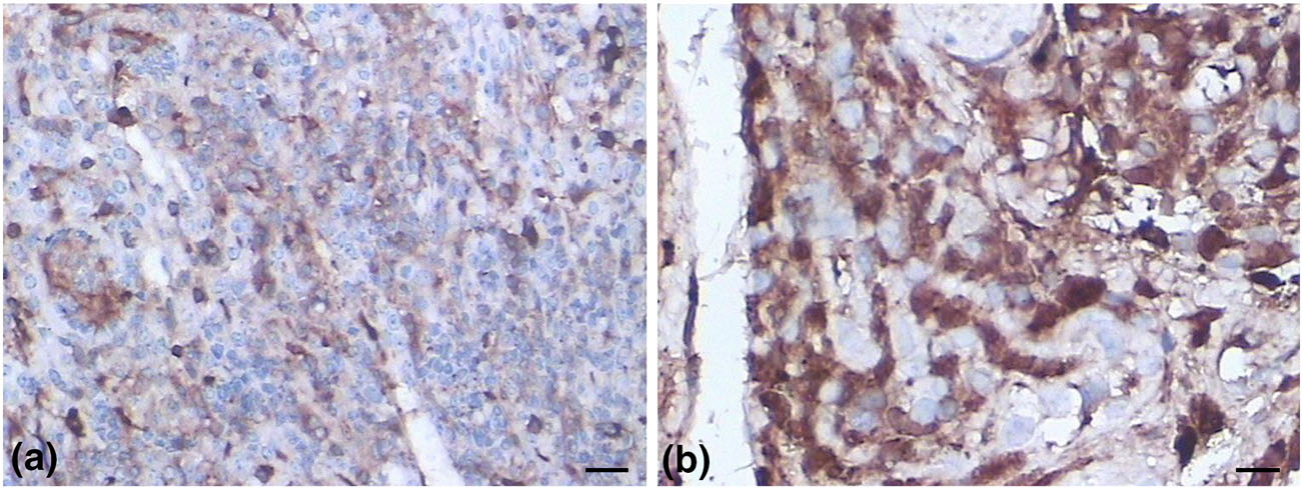
Immunostained samples for Ki‐67 labelling index in the kidney of the mice infected with Candida albicans. (a) Ki‐67 labelling index of mouse died within 30 days (3%); (b) Ki‐67 labelling index of mouse died after 30 days (40%; scale bar = 50 μm) [Colour figure can be viewed at wileyonlinelibrary.com]

Five‐month survival rates of all mice are listed in Table 4. Using the sources of isolation as parameter, strains could be divided in lung cancer, HIV/AIDS, VVC, and asymptomatic individuals. On the basis of strain sources and analysed by log‐rank test and Gehan–Breslow–Wilcoxon test, overall differences in survival were highly significant (*p* < .05). Isolates from lung cancer and AIDS proved to have higher virulence than VVC strains (Figure 2). Figure 3a shows histopathology of kidney derived from mice died within 48 days. Flat urothelial hyperplasia comprised an increased number of cell layers with few or no significant cytological abnormalities; fungal cells were predominantly in the hyphal form. Polarity was preserved, and none to minimal variation in architectural and nuclear features was noted. Figure 3b shows histopathology of kidney derived from a mouse that died after 67 days. Inflammation and the atypical epithelial dysplasia were observed in some infected mice; variations in nuclear polarities, sizes, shapes, and chromatin patterns occurred. Nuclei were uniformly enlarged with mild differences in shape, contour, and chromatin distribution, leading to a diagnosis of non‐invasive, low‐grade (ICD‐O code: 8130/21) urothelial carcinoma (John, Guido, Jonathan & Isabell, 2004). Mitoses were frequent, sometimes atypical, and occurred at any level including the surface. Figure 3c shows a predominant pattern of disorder with easily recognizable variations in architectural and cytological features even at low magnification. Marked variations in nuclear polarity, size, and shape and pattern of chromatin were recognized. Nuclei often showed pleomorphism with moderate to marked variation in size and with irregular chromatin distribution. Nucleoli were prominent. This led to a diagnosis of non‐invasive urothelial carcinoma, high grade (ICD‐O code: 8130/23; John et al., 2004). It is important to note that linear expression with occasional local interruption was observed. Table 1 shows carcinoma occurrence of mice infected with C. albicans strains. Abnormal cells were observed on pathologic sections of murine organs with C. albicans infection of 21 days, which was noted by the Ki67 labelling index of 3% (Figure 4a). Figure 4b shows C. albicans infection after 48 days; the Ki67 labelling index was 40%, leading to a diagnosis of non‐invasive urothelial carcinoma, high grade.

**Table 4:** Mortality of mice infected with Candida albicans strains

Group	Strain	CAI genotype	Source	Mice	Mortality10 days	Mortality21 days	Mortality5 months
1	GYF006	30–45	VVC	12	2 (16.6%)	6 (50.0%)	11 (91.6%)
2	GYZ249	21–21	Lung cancer	12	5 (41.6%)	9 (75.0%)	9 (75.0%)
3	ATCC 10231	Untested	Bronchopneumonia	12	3 (25.0%)	7 (58.3%)	9 (75.0%)
4	GYWu27	21–21	HIV/AIDS	12	3 (25.0%)	7 (58.3%)	12 (100.0%)
5	GYWu45	21–21	HIV/AIDS	12	2 (16.6%)	8 (66.7%)	12 (100.0%)
6	GYF76	32–46	VVC	12	1 (8.3%)	6 (50.0%)	11 (91.6%)
7	GYWu20	21–33	HIV/AIDS	12	3 (25%)	7 (58.3%)	12 (100.0%)
8	GYF091	30–46	VVC	12	2 (16.6%)	6 (50.0%)	12 (100.0%)
9	GYZ210	21–33	Lung cancer	12	4 (33.3%)	8 (66.7%)	11 (91.6%)
10	GYZ072	21–33	Lung cancer	12	5 (41.6%)	6 (50.0%)	11 (91.6%)
11	BK60	Untested	Normal	12	2 (16.6%)	5 (41.6%)	8 (66.7%)
12	S165	Untested	Normal	12	2 (16.6%)	6 (50.0%)	10 (83.3%)
13	1107	Untested	Normal	12	1 (8.3%)	4 (33.3%)	8 (66.7%)
14	Saline	‐	‐	12	0 (0.0%)	0 (0.0%)	0 (0.0%)

Abbreviation: VVC, vulvovaginal candidiasis.

## 4 DISCUSSION

In the current study, genotyping of C. albicans strains from VVC and non‐VVC disorders using microsatellite analysis of CAI and 25S rDNA revealed that most VVC strains possessed specific genotypes. Logistic regression analysis showed that, compared with other genotypes, the prevalent CAI genotypes of VVC have a high risk of OR (OR = 4.3). It shows that the risk of VVC in prevalent genotype was 4.3 times higher than that in other genotypes after controlling for age, marital, and sexual factors. CAI microsatellite genotyping has repeatedly been proven to be a powerful method for epidemiology of C. albicans (Bai, 2014). This approach revealed a significantly larger diversity than classical typing using 25S rDNA introns. The methods are not entirely congruent, indicating that, on the basis of criteria of genealogical concordance, recombination occurs in the analysed set of strains. However, 63 of the prevalent isolates from VVC (70.0%) with microsatellite types 30*–*45 and 32*–*46 and similar genotypes all belonged to 25S rDNA genotype A. This indicates the occurrence of expansion of closely related genotypes in a particular lineage.

Out of 57 microsatellite genotypes revealed in this study, 12 seemed to have a global distribution, because the same genotypes were reported in Europe (Kageyama et al., 2004). Thirty‐six genotypes were shared between Beijing/Tianjing, Guangdong, Guiyang, and Wuhan, representing northern, southern, western, and central regions of China, respectively (Bai, 2014; Fan et al., 2008; Ge et al., 2010). Seven novel genotypes were recognized in the current study, which seemed to have a local distribution.

Genotype CAI microsatellite patterns were also structured by association with clinical types. Profiles of systemic infections were found to differ significantly from those of VVC. The majority of randomly taken C. albicans strains associated with VVC belonged to the same or highly similar CAI genotypes. Most isolates from patients with lung cancer were recognized as CAI 21–33 and similar genotypes. In contrast, the overall pattern of strains originating from respiratory diseases and from AIDS patients was highly diverse. In respiratory disease, the diversity may be explained by local severe abuse of antibiotics to treat respiratory disorders, which has a negative impact upon the host's immune system and thus predisposes to fungal infection; the number of opportunistic infections in those patients is predicted to be high. In addition, the use of sputum samples rather than bronchoalveolar lavage fluid or blood may have led to the inclusion of commensal strains with deviating genotypes. AIDS patients, with 16 CAI types occurring in 23 strains, also seem to be infected easily by diverse C. albicans genotypes (Figure 1 and Table S1).

rDNA genotypes A and B versus C differ by absence and presence of a 25S rDNA‐based group I intron. C. albicans strains with relatively effective adherence properties are known to have higher virulence than non‐Albicans *Candida* species such as Candida krusei (Ferreira, Prado, Carvalho, Dias, & Dias, 2013). Genotype A seems to be more virulent than genotypes B and C (Sardi, Duque, Hofling, & Goncalves, 2012) and is dominant in VVC and in healthy oral mucosa of all age groups (Xu, Chen, & Li, 2008). In our study, all strains of the preponderant microsatellite genotypes from VVC (mainly 30*–*45, 32–46, and 30*–*46) belong to rRNA genotype A, which is consistent with current literature.

The hypothesis that strains differing in predilection for body sites might differ in virulence has been verified with animal inoculation experiments (Li et al., 2015). A progressive reduction of virulence was observed, with systemic isolates being more virulent than VVC strains (Figure 2), judged from the time of death after challenge and from pathologic expression in the infected mice.

We also showed differences in pathologic anatomy of various tissues after prolonged survival of *Candida* yeast cells. The Ki‐67 labelling indices of proliferating cells in mice that died after 3 weeks were mostly around 3%. A threshold Ki‐67 labelling index of >3% immune reactivity is termed “atypical adenoma,” which suggests a potential stimulus by *Candida* cells (Heaney, 2011a, 2011b; Šteňo et al., 2014). In mice died 2 months after inoculation, obviously non‐invasive urothelial carcinoma was observed (Dalgliesh et al., 2010), a disease associated with tuberous sclerosis that can result in kidney cancer (Kapoor, Sikri, Grover, Malhotra, & Sachdeva, 2014; Ramirez‐Garcia et al., 2016). A recent study supports the view that C. albicans infection may not just be a consequence of cancer but is an actively contributing cause as well causative agent (Nørgaard et al., 2013). A significant positive association has been reported of oral *Candida* carriage/infection and oral epithelial dysplasia/neoplasia (Ramirez‐Garcia et al., 2016). Since then, several studies have demonstrated that oral cancer and precancer lesions are frequently infected by *Candida* species. The most widely accepted hypothesis is that C. albicans produces nitrosamines, which are carcinogens that activate specific proto‐oncogenes that could trigger cancerous lesions (Krogh, 1990). In addition, alcohol dehydrogenase (ADH1) produced by C. albicans might metabolize alcohol and carbohydrates to acetaldehyde, which is carcinogenic and eventually induces tumour development. Possibly, C. albicans, Candida tropicalis, and C. parapsilosis produce more acetaldehyde than other *Candida* species, often exceeding carcinogenic levels (>100μM; Nieminen, Uittamo, Salaspuro, & Rautemaa, 2009).

C. albicans yeast cells are transported via the bloodstream into several tissues of the model animal, causing high levels of inflammation. The degree of damage to kidneys in inoculated animals was found to be more severe than to other organs, irrespective of genotype or clinical origin of the inoculated strain. Earlier publications also mentioned that *Candida* infection was less controlled in the kidney than in other organs (Li et al., 2015). Yeast cells penetrated through blood vessels into both cortex and medulla, causing an influx of neutrophils. Several authors mentioned an association between inflammation and cancer (Balkwill & Mantovani, 2001; Coussens & Werb, 2002). The inflammatory state enhances maintenance and promotion of tumour progression and the emergence of completely malignant phenotypes through neoplastic tissue remodelling, angiogenesis, metastasis, and inhibition of anticancer innate immune response (Wang, Arun, Friedman, Chen, & Van Waes, 2009). Specifically, IL‐18 and TNF‐α play a vital role in tumour adhesion and metastasis (Ramirez‐Garcia et al., 2011; Rodriguez‐Cuesta et al., 2010).

In the present experiment, non‐invasive urothelial carcinoma (high grade) was found in the experiment mice population. The mechanism that caused the appearance of this phenomenon is as yet unclear but requires close attention. We emphasize that there is a need not only to control *Candida* infection during cancer therapy, especially given the important role of this yeast in nosocomial infections, but also to find new therapeutics to suppress protumour effects by C. albicans.

Strains with rDNA genotype A have been shown to have decreased susceptibility to the antifungal agent flucytosine (McCullough, Clemons, & Stevens, 1999). Heterogeneity in resistance between strains is known to exist, but this was ascribed to geographic variation (Fan et al., 2008). Our strains of genotype A originating from VVC showed increased resistance to itraconazole but neither to flucytosine nor to other drugs tested. The correlation of the absence of a group I intron in the 25S rRNA with high levels of resistance to flucytosine was therefore not confirmed. The found deviations in drug resistance profiles of C. albicans genotype A may thus have been spread by local epidemics. The result of antifungal susceptibility tests showed that all of C. albicans strains in our study were susceptible to amphotericin B and there was a small difference in susceptibility to flucytosine and fluconazole; however, the strains from VVC were less susceptible to itraconazole than strains from non‐VVC infections. For some clinicians, the azoles have become the drug of choice for treatment of *Candida* infections, because of high bioavailability, extensive tissue penetration, long half‐life, convenient use, and good safety. Especially, the superficial infection VVC is more diverse than deep infection for local application. But the widespread overuse induces the emergence of drug‐resistant strains, resulting in a decline in susceptibility, when clinical medications do not follow the standard guidelines.

## CONFLICT OF INTEREST

None of the authors have conflict of interest.

## Supporting information

Table S1 Supporting information
